# LH Levels May Be Used as an Indicator for the Time of Antagonist Administration in GnRH Antagonist Protocols—A Proof-Of-Concept Study

**DOI:** 10.3389/fendo.2019.00067

**Published:** 2019-02-12

**Authors:** Minghui Liu, Shan Liu, Lingxiu Li, Peng Wang, Huanhuan Li, Yuan Li

**Affiliations:** Medical Center for Human Reproduction, Beijing Chao-Yang Hospital, Capital Medical University, Beijing, China

**Keywords:** assisted reproductive technology, controlled ovarian stimulation, LH level, GnRH antagonist, pregnancy outcomes

## Abstract

**Objective:** To investigate whether circulating LH levels could be used as an indicator for the timing of antagonist addition in GnRH antagonist protocol.

**Design:** Retrospective cohort study.

**Setting:** University-based hospital.

**Patients:** A total of 567 women stimulated with recombinant FSH monotherapy in a GnRH antagonist protocol were studied. Among them, 256 patients showed relatively low LH levels [highest LH level (LHmax) < 4 IU/L] during the entire ovarian stimulation process; 88 (Group A) and 168 patients (Group B) were stimulated without and with antagonist co-treatment, respectively. The remaining 311 patients had LHmax≥4 IU/L and were stimulated with a modified flexible antagonist protocol based on LH levels (Group C).

**Intervention(s):** Patients in Group B and C received antagonist during ovarian stimulation, whereas patients in Group A did not.

**Main outcome measure:** Clinical pregnancy rate and ongoing pregnancy rate.

**Results:** The clinical and ongoing pregnancy rates were significantly higher in group A than group B (69.3 vs. 54.7%, *P* = 0.03 and 62.5 vs. 48.2%, *P* = 0.04, respectively), but the primary outcome measures did not differ between groups B and C. There were no significant differences in terms of patient demographics, LH levels, total dosage of gonadotrophin, duration of stimulation, follicular output rate between groups A and B, and between groups B and C. Also, there were no significant differences in laboratory and clinical outcomes in pairwise group comparisons. No canceled cycles due to premature ovulation was reported among the treated patients.

**Conclusion:** LH levels may be used as an indicator for the time of antagonist addition. Patients with sustained low LH levels (LHmax<4 IU/L) during controlled ovarian stimulation (COS) might not require antagonist administration. Although further well-designed randomized controlled trials (RCTs) are needed to confirm our results, a novel treatment regimen based on LH measurements during COS might provide clinicians new insights about when to start antagonist administration in the GnRH antagonist protocol.

## Introduction

Over the past few decades, increasing knowledge of ovarian physiology and the possibility of accurately evaluating the ovarian reserve have progressively led to the individualized tailoring of ovarian stimulation for *in vitro* fertilization (IVF) treatment. During a controlled ovarian stimulation (COS), the suppression of the premature luteinizing hormone (LH) surge is essential for achieving satisfactory outcomes in assisted reproductive technology (ART). The protocols for COS generally use gonadotropin-releasing hormone (GnRH) agonists to prevent the premature rise of endogenous LH before follicular maturation (1). In recent years, GnRH antagonists have been shown to be useful for the rapid and reversible suppression of LH release. The use of the GnRH antagonist protocol, combined with the GnRH agonist trigger, and the freeze-all policy are recommended for the prevention of ovarian hyperstimulation syndrome (OHSS) (2–4). The starting day of GnRH antagonist administration in conventional GnRH antagonist protocols (i.e., both the fixed and flexible protocols) are mainly based on the day of ovarian stimulation, the diameter of the follicles, the estradiol levels, or a combination of these parameters (5). These protocols mainly focus on preventing an endogenous LH surge disregarding the LH levels during follicular growth. LH is essential for normal follicular development and oocyte maturation (6). In particular, LH can promote the proliferation and differentiation of theca cells for androgen secretion, which synergistically increases estrogen production (7). In the late follicular phase, LH helps to produce small amounts of progesterone, thereby promoting positive estrogen feedback, which is necessary for follicular development and maturation (8). Many studies have revealed the great importance of LH levels during COS in the follicular development and clinical outcome (9, 10). Fluctuations in LH levels during the follicular phase have a significant impact on morphological and functional changes of the oocyte and further affect its meiotic status and its ability to be fertilized (11).

Some researchers have proposed that there is a “LH clinical treatment window,” in which LH levels higher than the “LH ceiling” are associated with abnormal follicular development (12). GnRH antagonist protocols have been widely used in IVF treatments in recent years (13). By competitively binding to the pituitary GnRH receptor, antagonists can rapidly and effectively inhibit the LH levels, with no resulting “flare up” effect (14). Antagonists are flexible and easy to use and have a shorter action time than GnRH agonists. Notably, studies have found that elevated LH levels in the early follicular phase (early-onset LH peaks) had an adverse effect on pregnancy outcomes (15). Other studies found that when LH levels during COS are < 1/3 of the LH levels on the trigger day, the pregnancy rate decreases, which indicates that LH plays an important role in oocyte development (16).

The secretion and response of LH levels to antagonists vary widely from individual to individual (17). The purpose of the antagonist regimen is to suppress early-onset LH peaks and, subsequently, early-onset ovulation (14). However, in some patients, the endogenous LH levels are not enough to fully support the development of the follicles; these patients might not need an antagonist regimen (18). Although some studies have shown that serum LH levels should be between 1.2 and 5.0 IU/L for optimal development follicle (11, 19), there is a lack of clear recommendations regarding the use of GnRH antagonists based on serum LH levels during COS.

As a regulator of LH levels, we hypothesize that the addition of GnRH antagonist might be tailored according to LH levels during ovarian stimulation. To explore this hypothesis, we have used a more flexible antagonist protocol for selected patients based on serum LH levels routinely assessed during COS. In our settings, a serum LH of 4 IU/L is used as the cutoff value to decide whether or not GnRH antagonists are administered. This threshold is based on our observations using frequent LH measurements during COS. Accordingly, we found that some patients with low LH levels (<4 IU/L) throughout COS had no LH surge. Considering that the administration of a GnRH antagonist would further decrease LH levels, we decided to stimulate some of our patients with LH levels below 4 IU/L without antagonist co-treatment.

Thus, our study aimed to determine whether LH could be used as an indicator for the timing of antagonist addition in GnRH antagonist protocol. If our hypothesis proved to be true, new protocols might be established to more effectively control the LH levels, and potentially improve the efficacy of IVF treatment, especially for those with low LH levels and no LH surge during COS.

## Materials and Methods

### Study Design

**Figure 1:**
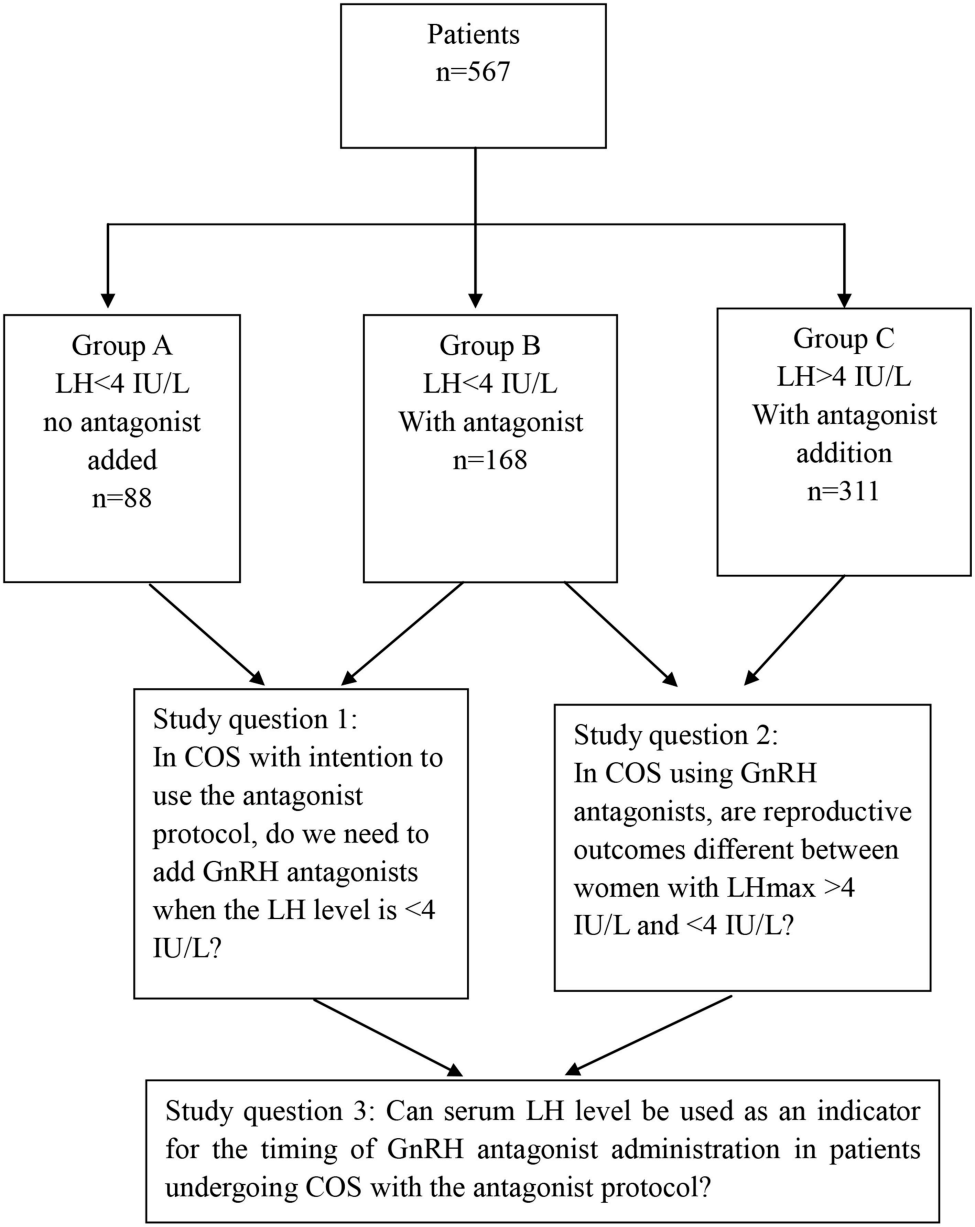
Study design.

This is a retrospective, observational single-center cohort study conducted at the Center of Reproductive Medicine, Beijing Chao-Yang Hospital from January 1, 2016 to August 1, 2018. The study design and study's questions are shown in Figure 1. All patients provided informed consent. Furthermore, the study was approved by the Institutional Review Board of our hospital.

### Patients

Patients were included if they fulfilled the following criteria: (i) age <40 years; (ii) baseline serum FSH levels <12 IU/L; and (iii) no uterine polyp, adhesion, Mullerian malformations, endometriosis, adenomyosis, or adnexal (hydrosalpinx) abnormalities. Poor responders identified according to Bologna criteria were excluded. A total of 567 women were included, which were divided into three groups according to LH levels during COS and whether GnRH antagonists were prescribed, as below.

Group A: 88 patients, sustained low LH levels during COS [the highest LH level (LHmax) was <4 IU/L], no GnRH antagonist given;

Group B: 168 patients, sustained low LH levels during COS (LHmax <4 IU/L); conventional flexible GnRH antagonist co-treatment given;

Group C: 311 patients, LHmax ≥4 during COS; modified flexible GnRH antagonist co-treatment based on LH measurements given.

### Stimulation Protocol

An individualized dose of 150–300 IU rFSH (Gonal-f®; Merck) was administered daily for ovarian stimulation from day 3 of the menstrual cycle. Four to five days later, the serum levels of estradiol (E2, pg/ml) and LH (IU/L) were measured, and an ultrasound was performed. Gonadotropin dosage was adjusted according to hormone levels and follicle development.

Hormone analyses were performed at least four times during COS, as follows: (i) day 1 of stimulation; (ii) 4–5 days after stimulation initiation; (iii) 2 days later, i.e., 6–7 days after initiation; (iv) the day of triggering. Additionally, morning urine LH level testing was done by the patient themselves; if a positive result was observed, blood was taken for LH measurement immediately.

Since LH levels were monitored regularly during the whole COS period, we used a modified flexible antagonist protocol based on LH levels in some patients. Briefly, from day 5 of stimulation, no antagonist was administered if the LH level was lower than 4 IU/L. By contrast, if LH levels were 4 IU/L or higher, 0.25 mg cetrorelix acetate (Cetrotide®; Merck) was given daily for 2 days until the next blood test. The decision to continue antagonist co-treatment was based on subsequent LH results >4 IU/L until trigger day. Patients in Group A and C were stimulated using the modified protocol described above. By contrast, patients in Group B were stimulated using the conventional flexible antagonist protocol; in these patients, the antagonist was given from the day when estradiol levels reached 400 pg/ml or the dominant follicle reached 14 mm in diameter until trigger day despite LHmax <4 IU/L.

Triggering of final oocyte maturation was carried out when the leading follicle reached 18–20 mm or more than three follicles measuring 16 mm were observed. For this, 0.2 mg triptorelin (Decapeptyl®; Ferring) plus 1,000–2,000 IU hCG (recombinant human chorionic gonadotrophin, Ovitrelle®, Merck) were administered. Oocyte retrieval was performed 36 h later.

### Embryo Transfer and Luteal Phase Support

Fresh embryo transfers were performed 3 days after oocyte retrieval (D3). Vaginal progesterone gel (90 mg/d, Crinone®, Merck) and oral dydrogesterone (10 mg twice a day, Duphaston®, Abbott) were administered for luteal phase support. Freeze-all procedures were performed in patients with a high risk of developing OHSS, as well as in patients with serum progesterone >1.5 ng/ml during COS and those with an endometrial thickness <7 mm. In such cases, two good quality embryos were vitrified on day 3, and the remaining embryos were cultured for 2–3 more days for blastocyst vitrification.

In the first frozen-thawed transfer cycle, only D3 embryos were transferred. Frozen-thawed embryo transfers were performed in any of the following cycle using either an artificial or natural cycle. Briefly, for artificial endometrial preparation, patients received oral estradiol valerate (Progynova®; Bayer) 6 mg/day from the second day of the menstrual cycle for 10–12 days. Endometrial thickness was assessed by vaginal ultrasonography. When endometrial thickness reached ≥8 mm, progesterone was given as in fresh cycles. Embryo transfer was performed on the 4th day of progesterone administration. For natural cycles, follicle development was monitored from day 12th of the menstrual cycle. Embryo transfer was performed 3 days after ovulation. Serum hCG levels were tested 12–14 days after the embryo transfers. Once the patients were pregnant, the luteal phase support continued until ~9–10 weeks of gestation.

### Outcome Variables

Baseline characteristics of the patients, including age, BMI (kg/m^2^), the duration of infertility, infertility factors, basal hormone levels, the antral follicle count, the dosage of GnRH antagonist, stimulation parameters, and laboratory and clinical outcomes were collected and compared among groups. Concerning the clinical outcomes, pregnancy results after the first embryo transfer cycle with the best two D3 cleavage embryos, either fresh, or frozen cycle, were analyzed.

The primary outcome (ongoing pregnancy rate) was defined as any delivery of a live-born child, as well as an ultrasonographical viable pregnancy beyond 12 weeks of gestation for those who had not yet delivered until the time of study completion. The FORT was defined as the ratio of the number of 16–22 mm preovulatory follicles (PFC) to the number of 3–8 mm antral follicles (AFC) on the third day of the menstrual cycle. The clinical pregnancy rate was confirmed by the presence of a gestational sac via an ultrasound. Early pregnancy loss was defined as a positive serum hCG test without ultrasound evidence of a gestational sac or a gestational sac without a fetal heartbeat.

### Statistical Analysis

Statistical analyses were performed using the Statistical Package for the Social Sciences (SPSS, version 22.0). Data were expressed as the mean ± standard deviation (SD). The independent samples *t*-test and the Mann–Whitney *U*-test were used for comparing continuous variables with normal and non-normal distributions, respectively. Frequencies were expressed in percentage (%) and were compared using a chi-square test or Fisher's exact test. A *P* < 0.05 was considered statistically significant.

## Results

### Baseline Data and Cycle Characteristics Between Groups A and B

A total of 256 patients had LH levels below 4 IU/l (LHmax <4) during the ovarian stimulation process. Of these, 88 patients did not use any antagonist (Group A, LHmax <4, no antagonist), and 168 patients used the flexible antagonist protocol (Group B, LHmax <4, antagonist administration). The demographics of these patients are presented in Table 1. The patient characteristics were not significantly different between the two groups, and there were no significant differences in the laboratory outcomes. The clinical pregnancy rates (69.3 vs. 54.7%) and ongoing pregnancy rates (62.5 vs. 48.2%) were significantly higher in Group A than in Group B (Table 2). No cycle cancellation due to an unexpected premature ovulation was reported among patients of groups A and B.

**Table 1:** Baseline characteristics and laboratory outcomes between Groups A and B.

	**Group A**	**Group B**	***P*-value**
Patients, *n*	88	168	
Age (years)	31.7 ± 4.3	31.9 ± 3.8	0.62
Duration of infertility (years)	3.0 ± 2.3	3.0 ± 2.4	0.99
BMI (kg/m^2^)	22.9 ± 3.6	23.1 ± 3.9	0.84
Antral follicle count	13.3 ± 6.2	14.7 ± 5.9	0.08
Total gonadotrophin (IU)	2456.6 ± 696.7	2304.8 ± 691.4	0.10
Total stimulation duration (days)	9.9 ± 1.5	9.7 ± 1.3	0.20
LHmax (IU/L)	2.7 ± 1.3	2.8 ± 0.8	0.50
Baseline E_2_ (pg/mL)	46.0 ± 27.2	47.0 ± 21.3	0.76
Baseline FSH (IU/L)	5.9 ± 2.4	6.1 ± 2.2	0.54
Baseline LH (IU/L)	3.2 ± 1.5	3.8 ± 1.7	0.20
Endometrial thickness on hCG day	10.0 ± 2.2	9.9 ± 2.2	0.90
FORT	0.8 ± 0.4	0.8 ± 0.4	0.07
Oocytes retrieved, *n*	14.3 ± 8.5	15.0 ± 7.3	0.51
MII oocytes, *n*	11.2 ± 7.9	11.9 ± 6.5	0.49
2PN zygotes, *n*	9.6 ± 6.6	9.5 ± 5.5	0.88
High quality embryos, *n*	4.3 ± 3.1	4.8 ± 2.2	0.24

*BMI, body mass index; LHmax, highest LH levels during controlled ovarian stimulation; E2, estradiol; FSH, follicle stimulating hormone; LH, luteinizing hormone; hCG, human chorionic gonadotropin; MII, metaphase II; 2PN, two-pronuclei; FORT, follicular output rate*.

**Table 2:** Reproductive outcomes between Groups A and B.

	**Group A**	**Group B**	***P*-value**
Patients, *n*	88	168	
Positive hCG test, *n* (%)	63/88 (71.6%)	102/168 (60.7%)	0.08
Clinical pregnancy, *n* (%)	61/88 (69.3%)	92/168 (54.7%)	0.03
Ongoing pregnancy, *n* (%)	55/88 (62.5%)	81/168 (48.2%)	0.04
Early pregnancy loss, *n* (% of positive hCG)	8/63 (12.7%)	21/102 (20.6%)	0.20
Canceled cycles due to premature LH surge, *n*	0	0	–

### Baseline Data and Cycle Characteristics Between Group B and Group C

As previously mentioned, GnRH antagonists were administered in Group B patients using a flexible protocol despite LHmax <4 IU/L. In Group C, all patients had LHmax ≥4 IU/L and therefore the antagonist was added. The baseline characteristics and laboratory outcomes were not statistically different between patients of Groups B and Group C (Table 3). Likewise, no significant differences were noted in the outcome measures between the groups (Table 4). Furthermore, no cycle was canceled due to an unexpected premature ovulation.

**Table 3:** Baseline characteristics and laboratory outcomes between Groups B and C.

	**Group B**	**Group C**	***P*-value**
Patients, n	168	311	
Age (years)	31.9 ± 3.8	32.1 ± 3.8	0.06
Duration of infertility (years)	3.0 ± 2.4	3.1 ± 2.3	0.67
BMI (kg/m^2^)	23.1 ± 3.9	22.5 ± 4.1	0.17
Antral follicle count	14.7 ± 5.9	15.8 ± 8.5	0.08
Total gonadotrophin (IU)	2304.8 ± 691.4	2389.9 ± 800.8	0.22
Total stimulation duration (days)	9.7 ± 1.3	9.6 ± 1.4	0.36
LHmax (IU/L)	2.8 ± 3.8	7.5 ± 3.8	0.00
Baseline E2 (pg/mL)	47.0 ± 21.3	49.2 ± 22.5	0.30
Baseline FSH (IU/L)	6.1 ± 2.2	6.2 ± 2.3	0.40
Baseline LH (IU/L)	3.8 ± 1.7	3.9 ± 1.5	0.15
Endometrial thickness on hCG day	9.9 ± 2.2	10.1 ± 2.1	0.49
FORT	0.8 ± 0.4	0.8 ± 0.6	0.40
Oocytes retrieved, n	15.0 ± 7.3	14.7 ± 7.7	0.69
MII oocytes, n	11.9 ± 6.5	11.7 ± 6.7	0.80
2PN zygotes, n	9.5 ± 5.5	9.3 ± 5.6	0.66
High quality embryos, n	4.8 ± 2.2	4.4 ± 2.5	0.16

**Table 4:** Reproductive outcomes between Groups B and C.

	**Group B**	**Group C**	***P*-value**
Patients, *n*	168	311	
Positive hCG test, *n* (%)	102/168 (60.7%)	177/311 (56.9%)	0.42
Clinical pregnancy, *n* (%)	92/168 (54.7%)	156/311 (50.2%)	0.34
Ongoing pregnancy, *n* (%)	81/168 (48.2%)	145/311 (46.6%)	0.77
Early pregnancy loss, *n* (% of positive hCG)	21/102(20.6%)	32/177(18.1%)	0.61
Canceled cycles due to premature LH surge, *n*	0	0	–

## Discussion

In this retrospective cohort study, we explored the clinical utility of measuring LH levels during COS with FSH monotherapy to determine the need of adding GnRH antagonists. We found that among patients with low endogenous LH levels (LHmax <4 IU/L) during COS, the clinical pregnancy rate and ongoing pregnancy rate were significantly higher in patients who did not use GnRH antagonists than those who did. Notably, no cycle was canceled due to premature LH surge in patients with LHmax <4 IU/L who did not use antagonists. Furthermore, among women who used GnRH antagonists during COS, there were no differences in reproductive outcomes as well as cycle cancellation rates irrespective of whether or not the LHmax was ≥4 IU/L or <4 IU/L. Our results suggest that LH levels could be used as an indicator for the need of antagonist addition in the GnRH antagonist protocol among women undergoing COS for IVF/ICSI.

The existing clinical evidence has indicated two essential aspects concerning the use of GnRH antagonists in association with COS in the overall IVF-ICSI population, namely, (1) GnRH antagonists competitively block pituitary gland GnRH receptors and induce a rapid gonadotropin suppression (20), and (2) a threshold level of endogenous LH is required for adequate follicular development and oocyte maturation, and severe suppression of mid-follicular LH levels is associated with poor reproductive outcomes (12). Our observations are therefore consistent with a possible detrimental effect of LH suppression by GnRH antagonists in women with decreased LH levels during COS. In our study, LH levels of all patients in groups A and B remained low (LHmax <4 IU/L) throughout COS. With administration of the GnRH antagonist, the LH levels are expected to decrease further, which in the present study adversely affected pregnancy outcomes.

Since its introduction many studies have sought to determine the best day to starting GnRH antagonist administration (21, 22). The first GnRH antagonist protocol developed was the fixed protocol, which is based on administration of GnRH antagonist from days 5 or 6 onwards (22). Subsequently, the flexible protocol was introduced to reduce the number of GnRH antagonist injections (23). While preferences vary, both protocols appear to be used widely (21). However, most studies evaluating technical aspects of GnRH antagonist protocols mainly focus on day of gonadotropin stimulation or diameter of the follicles rather than LH levels to guide GnRH antagonist administration. In our clinical practice, however, we have observed that some IVF/ICSI patients undergoing COS show LH levels below 4 IU/L during the whole stimulation period. Since previous studies demonstrated that markedly suppressed LH might have an adverse impact on cycle outcomes and that antagonist administration further decrease LH levels (10, 16), we explored the possibility of abstaining from using GnRH in such patients.

Our results indicate that women with LH levels lower than 4 IU/L during COS with FSH monotherapy do not require addition of antagonists for LH suppression. These patients had similar cycle outcomes to counterparts who received antagonists, but clinical and ongoing pregnancy rates were significantly higher in the former. Notably, peak LH levels during COS, FORT, cycle cancellation due to premature ovulation, and embryonic outcomes did not differ between these groups. The mean peak LH levels were 2.7 and 2.8 IU/L in groups A and B, respectively, thus indicating that these patients had decreased endogenous LH secretion.

We speculate that a possible reason explaining the poorer pregnancy outcomes among women with LHmax < 4 IU/L who used the antagonist co-treatment was a further suppression of LH levels. Previous reports have shown poor pregnancy outcomes in patients who have low LH levels, or who experience a sharp fall in LH levels from baseline levels (16). In one report using mild ovarian stimulation protocol with a GnRH antagonist, the pregnancy rate and implantation rate were significantly lower when the serum LH level was less than one-third of baseline levels at the time of hCG injection (16). Lahoud et al. found that a relative reduction in mid-follicular LH concentrations during GnRH agonist cycles leads to lower live birth rates (24). Our study concurs with these observations of reduced clinical and ongoing pregnancy rates possibly caused by LH changes due to antagonist addition.

The aim of the GnRH antagonist administration in IVF cycles is to prevent an untimely LH surge and premature luteinization. Indeed, LH elevations are reported to occur in 1.4–35% of patients undergoing COS with the GnRH antagonist protocol (25). However, our patients with LHmax <4 IU/L had no signs of follicular luteinization or early ovulation despite abstaining from using GnRH antagonists. Moreover, the laboratory and clinical parameters were not significantly different from counterparts who used antagonists, thus support the concept that the optimal starting day of antagonist administration can be determined by LH levels.

In our study, both patients in groups B and C were treated with antagonists. While the conventional flexible antagonist protocol was used in Group B patients (LHmax <4 IU/L), Group C patients were treated with the modified LH-based antagonist protocol. In the latter, administration of antagonists was based on detection of LH levels >4 IU/L during serial hormone assessment during COS. We found no differences between groups B and C concerning laboratory and clinical data, nor did we find any statistical significant difference in pregnancy outcomes, with ongoing pregnancy rates of 48.2 and 46.6% in groups B and C, respectively. Thus, our results suggest that the LH-based antagonist protocol is not inferior to the conventional flexible protocol.

Although the antagonist was given when LH levels were higher than 4 IU/L in group C patients, there were no canceled cycles due to an early ovulation event before retrieval. We hypothesize that an individualized starting day for administration of the GnRH antagonist may promote an optimal ovarian response. By starting GnRH antagonist administration when LH levels reached 4 IU/L, we were able to avoid the LH surge, and apparently the stimulation outcomes were not adversely affected.

GnRH antagonists directly affect endogenous LH levels, which are associated with oocyte development and COS outcomes. We believe that LH levels during COS need to be considered for a better ART outcome (26). On the one hand, higher LH levels in the follicular phase coincide with compromised outcomes of natural and stimulated cycles (27). On the other hand, low LH levels relate to increased early pregnancy loss in IVF/ICSI (28). As regulators of LH levels, use of GnRH antagonists should take LH levels during COS into account for a better individualization of the stimulation protocol.

Along these lines, among patients with low LH level during COS, administration of antagonist might further decrease the LH levels and adversely affect reproductive outcomes. Our data is consistent with the above-mentioned as patients in group A achieved higher pregnancy outcomes than those in group B. Using the conventional flexible antagonist protocol, clinicians will not be able to identify such patients. However, regular LH monitoring might identify patients with sustained low LH levels during COS who do not require antagonist co-treatment. Lastly, our data also showed that the use of GnRH antagonists only when the LH level was over 4 IU/L did not increase cycle cancellation rate. In such patients, cycle outcomes were similar than those using the conventional flexible antagonist protocol.

The present study has several limitations. First, it is a retrospective analysis with the inherent limitations of a study of that nature. Besides, the sample size is relatively small. Second, we chose 4 IU/L as the LH cut-off based on our previous clinical observations; however, we cannot affirm that this is the best discriminatory cut-off value. Third, we did not include a comparison group involving women with normal LH levels and no antagonist. However, in our practice, we do not abstain from prescribing GnRH antagonists in these patients owing to high risk of premature ovulation and cycle cancellation. Fourth, we did not explore the use of LH supplementation during COS in patients with LHmax < 4 IU/L. Use of gonadotropins with LH activity might overcome the LH deficiency and therefore antagonist co-treatment might be required, a hypothesis that deserves further investigation. As a proof of concept study, we have demonstrated the clinical utility of LH measurement in IVF/ICSI patients undergoing COS with the antagonist protocol and FSH monotherapy. Further prospective randomized controlled trials (RCTs) are needed to confirm the validity of our observations. Currently, two multi-center RCTs on the matter concerned have been registered in chictr.org.cn and are now under way, in which 2,000 women are expected to be enrolled.

## Conclusion

A retrospective study was conducted to explore whether the use of antagonists might be based on serum LH levels. We found that among patients undergoing COS with FSH monotherapy, the use of a modified GnRH antagonist protocol based on LH determinations provided comparable results to the conventional flexible antagonist protocol. Moreover, our data suggest that patients with sustained low LH levels during COS do not require antagonist co-treatment. Our proposed treatment regimen can guide provided us with a new perspective about the starting day of antagonist administration in the GnRH antagonist protocol. LH levels may be used as an indicator for the time of antagonist addition. The clinical efficacy of this approach and the best discriminatory LH cut-off values require further confirmation in prospective randomized trials.

## Ethics Statement

All patients provided written informed consent to participate in the study, which was conducted in accordance with the Declaration of Helsinki. The research project was approved by the Ethics Committee of the Beijing Chao-Yang Hospital, Capital Medical University.

## Author Contributions

ML and SL: data collection and statistical analysis of the data; concepted the analysis and wrote the manuscript. YL: supervision of the study concept and review of manuscript. LL, PW, and HL: involved in patient's treatment and review of manuscript. All authors contributed to study design, and critical discussions.

### Conflict of Interest Statement

The authors declare that the research was conducted in the absence of any commercial or financial relationships that could be construed as a potential conflict of interest.
